# Live imaging of developing mouse retinal slices

**DOI:** 10.1186/s13064-018-0120-y

**Published:** 2018-09-15

**Authors:** Anthony P. Barrasso, Shang Wang, Xuefei Tong, Audrey E. Christiansen, Irina V. Larina, Ross A. Poché

**Affiliations:** 1 Department of Molecular Physiology and Biophysics, Baylor College of Medicine, Houston, TX 77030 USA; 2 Program in Developmental Biology, Baylor College of Medicine, Houston, TX 77030 USA; 3 Program in Integrative Molecular and Biomedical Sciences, Baylor College of Medicine, Houston, TX 77030 USA

**Keywords:** Live imaging, Mouse retinal progenitor cells, Interkinetic nuclear migration, *Cyclin D1*, Horizontal neurons

## Abstract

**Background:**

Ex vivo, whole-mount explant culture of the rodent retina has proved to be a valuable approach for studying retinal development. In a limited number of recent studies, this method has been coupled to live fluorescent microscopy with the goal of directly observing dynamic cellular events. However, retinal tissue thickness imposes significant technical limitations. To obtain 3-dimensional images with high quality axial resolution, investigators are restricted to specific areas of the retina and require microscopes, such as 2-photon, with a higher level of depth penetrance. Here, we report a retinal live imaging method that is more amenable to a wider array of imaging systems and does not compromise resolution of retinal cross-sectional area.

**Results:**

Mouse retinal slice cultures were prepared and standard, inverted confocal microscopy was used to generate movies with high quality resolution of retinal cross-sections. To illustrate the ability of this method to capture discrete, physiologically relevant events during retinal development, we imaged the dynamics of the *Fucci* cell cycle reporter in both wild type and *Cyclin D1* mutant retinal progenitor cells (RPCs) undergoing interkinetic nuclear migration (INM). Like previously reported for the zebrafish, mouse RPCs in G1 phase migrated stochastically and exhibited overall basal drift during development. In contrast, mouse RPCs in G2 phase displayed directed, apical migration toward the ventricular zone prior to mitosis. We also determined that *Cyclin D1* knockout RPCs in G2 exhibited a slower apical velocity as compared to wild type. These data are consistent with previous IdU/BrdU window labeling experiments on *Cyclin D1* knockout RPCs indicating an elongated cell cycle. Finally, to illustrate the ability to monitor retinal neuron differentiation, we imaged early postnatal horizontal cells (HCs). Time lapse movies uncovered specific HC neurite dynamics consistent with previously published data showing an instructive role for transient vertical neurites in HC mosaic formation.

**Conclusions:**

We have detailed a straightforward method to image mouse retinal slice culture preparations that, due to its relative ease, extends live retinal imaging capabilities to a more diverse group of scientists. We have also shown that, by using a slice technique, we can achieve excellent lateral resolution, which is advantageous for capturing intracellular dynamics and overall cell movements during retinal development and differentiation.

**Electronic supplementary material:**

The online version of this article (10.1186/s13064-018-0120-y) contains supplementary material, which is available to authorized users.

## Background

The mouse retina is a proven model system in which to understand the cellular and molecular mechanisms driving mammalian retinogenesis and the pathophysiology of retinal diseases [3, 17, 48]. This feature is largely due to several technical advantages such as the relative ease of utilizing Cre-loxP technology, plasmid electroporation, and viral transduction to perform in vivo genetic loss-of-function, gain-of-function, and lineage tracing experiments [8, 26, 27, 42]. Additionally, the embryonic and early postnatal mouse retina is amenable to ex vivo manipulation and cultured retinae retain many features of normal development [6, 12, 26, 33]. These methods allowed researchers to extend gene transfer and cell labeling approaches to discrete cell types and developmental time points. Rodent retinal explants also have the added advantage of allowing for time-lapse microscopy to directly monitor developmental processes such as retinal progenitor cell (RPC) interkinetic nuclear migration (INM) and cell cycle progression, as well as neuron migration and morphogenesis [18, 33].

To date, live fluorescent microscopic imaging of the rodent retina has been mostly limited to whole mount explants [6, 7, 14, 18, 20, 21, 23, 33, 39, 51, 52]. While much has been learned using this approach, significant technical hurdles remain. To image through the entire thickness of the retinal explant, one must account for the attenuation of light within deeper layers, a problem that is particularly true if researchers wish to capture events, such as INM, that occur along the radial axis. As a solution, many have turned to brighter and far-red shifted fluorescent reporters as well as imaging modalities such as 2-photon microscopy, which allow for deeper imaging with less photo-toxicity. However, specific reporters, such as fluorescent fusion proteins, may not be sufficiently bright. Furthermore, 2-photon microscopy may not be available to a particular researcher or the demand on institutionally shared equipment may make lengthy time-lapse experiments logistically impossible or cost prohibitive.

Here we present a straightforward method to perform living imaging of developing mouse retinal slice cultures for at least 24 h. This approach is designed to circumvent the need for 2-photon microscopy, which makes our approach more feasible to a wider array of scientists interested in studying mouse retinogenesis. Furthermore, we demonstrate that our slice preparation allows for excellent lateral resolution of the entire retinal cross-sectional area by confocal microscopy. This is a significant advantage for capturing intracellular dynamics and overall cell movements during retinal development and differentiation. To verify that live imaging of retinal slice cultures is capable of capturing normal retinal developmental processes, we have performed several proof-of-concept experiments. First, we analyzed INM patterns among RPCs and showed migration features in common with previous findings in zebrafish. Next, we explored the consequence of *Cyclin D1* loss on RPC INM rate and trajectory and found that mutant RPC apical migration was slower than controls. Finally, using horizontal cells (HCs) as an example, we determined that our protocol is sufficient for monitoring post-mitotic retinal neuron dynamics.

## Methods

### Retinal slice culture preparation (see Additional file 1 for detailed materials list)

#### Agarose and culture media preparation

We prepared 6.5% (40 mL) and 1.5% (30 mL) low melting agarose solutions in DMEM/F12 media without phenol red. 1000 mL and 500 mL glass beakers were used to make a double boiler on a hotplate in which the agarose was melted. Once the agarose is fully melted and appears clear, it can be kept in a 37 °C water bath until use. Under a tissue culture hood, DMEM/F12 complete culture media was prepared with 10% FBS, 1% Pen/Strep, and Insulin (5 μg/ml), and was warmed to 37 °C prior to use.

#### Dissection and embedding

To prepare for retinal dissections, a heating pad was warmed in a microwave for 2.5 min. 3 mL of culture media (warmed to 37 °C) was transferred into several 35 mm petri dishes and placed on the heating pad to keep the media warm. For all early postnatal time points, live pups were collected directly from the breeding cage and individuals with the desired fluorescent reporters were selected using a Zeiss Stemi 2000 stereomicroscope equipped with a Nightsea fluorescent adaptor. Fluorescent postnatal pups were sacrificed by CO_2_ inhalation followed by decapitation and skinning of the head. The skin tissue was reserved for PCR-based confirmation of genotypes. The eyes were carefully enucleated with a curved, serrated Graefe forceps and placed in the 35 mm petri dish with warm DMEM/F12 and maintained on the heating pad.

For retinal dissections, a plastic transfer pipette with a cut (widened) tip was used to transfer individual eyes to a fresh 35 mm petri dish containing DMEM/F12 at 37 °C and visualized under a stereomicroscope. With a pair of sharp #5 fine forceps, a hole was poked in the limbus of the eye and the scleral, retinal pigment epithelial, iris, and corneal tissues were carefully peeled away from the eye thereby leaving behind an intact retinal cup and lens. Next, the surface of the lens center was held by one sharp #5 forceps while a second forceps was used to gently pull the lens and any attached hyaloid vessels away from the retina. If significant hyaloid vasculature within the retinal cup persisted, it was carefully removed. Once an intact retinal cup was isolated, it was transferred with plastic transfer pipette to a new dish of fresh DMEM/F12 on the heating pad. This process was repeated until all of the retinal cups were collected. Care was taken to minimize rips in the retinal tissue and the dissections were performed as quickly as possible.

Once all retinae were dissected, they were embedded in the melted 6.5% agarose. Using a plastic transfer pipette, a Tissue-Tek® cryomold (10 mm X 10 mm X 5 mm) was filled halfway with 6.5% agarose. Using a fresh cut plastic transfer pipette, an isolated retinal cup was transferred from the media to the agarose within the cryomold. Care was taken to transfer as little media as possible to the agarose. Using #5 forceps, the retinal cup was oriented with the retinal ganglion cell layer facing up. Any residual media was carefully wicked away with a Kimwipe and additional 6.5% agarose was added to fill the mold. In order to solidify, the agarose was left at room temperature for 10 min.

#### Vibratome sectioning, agarose slice mounting, and culture

The vibratome buffer tray was filled with sterile 1X PBS and half of a double-edged razor blade was secured to the knife holder. Once the agarose solidified, it was removed from the cryomold and a single edge razor blade was used to trim the agarose block so that a slice would fit the dimensions of the 10 mm diameter glass coverslip mounted to a glass bottom dish. To make retinal cross-sections, the agarose cube containing the retina with the ganglion cell layer face up, was rotated 90 degrees and mounted to the vibratome specimen disc with super glue. With the vibratome frequency and speed both set to 5, 200 μm agarose slices were obtained and floated onto a paintbrush for transfer onto a glass bottom culture dish.

For the slice transfer, enough complete culture media (roughly 100 μL) was added to the cover slip of the glass bottom dish so that a stable bubble formed in the shallow well. The agarose slice floated onto the paintbrush and was carefully transferred to the culture media bubble. A P200 pipette was used to remove as much of the media as possible resulting in the retinal slice making direct contact with the glass of the dish. Next, using a plastic transfer pipette, the agarose slice was covered with a drop of warm 1.5% agarose media. Once this solidified, the entire bottom of the dish was filled with a thin layer (roughly 2 mL) of 1.5% agarose media that was then allowed to solidify. Finally, 1.5 mL of complete culture media was added on top of the agarose layer, and the culture was immediately transferred to a tissue culture incubator set to 37 °C and 5% CO_2_ until we were ready to image.

### Retinal immunofluorescence

Eyes were harvested from mice and the retina and lens were isolated as described above. The retina and lens were fixed in 4% paraformaldehyde at 4 °C for 1 h. The tissues were washed with 1X PBS, incubated overnight in 15% sucrose solution (Sigma-Aldrich S9378) at 4 °C and then transferred to 30% sucrose at 4 °C for an additional night. The tissue was then embedded in OCT compound (Sakura 4583) on dry ice and cryosectioned into 20 μm sections. Cryosections were washed with 1X PBS-T (1X PBS + 0.1% Triton-X 100), 3 times for 10 min each, and then blocked in 2% normal goat (Sigma-Aldrich G9023) or donkey serum (Sigma-Aldrich D9663) for 1 h at room temperature. Next, the tissue was stained with primary antibodies (diluted in blocking solution) overnight at 4 °C in a humid chamber. Then, the tissue was washed with 1X PBS for 3 times, 10 min each at room temperature and stained with secondary antibodies (in blocking solution) for 1 h at room temperature in a dark humid chamber. The tissue was again washed with 1X PBS 3 times for 10 min and a coverslip was mounted over the tissue with Fluoromount-G (Southern Biotech, #0100–01). Primary and secondary antibody concentrations are listed in Additional file 2.

### Assessment of cell death and proliferation within cultured retinal slices

To assess cell death, retinal slice cultures were briefly rinsed with 1X PBS and stained with the Zombie Red™ Fixable Viability Kit (Biolegend Ca# 423109). Cultures were incubated in the dye solution (1:500 in 1X PBS) for 1 h at 37 °C in the dark. The tissue was then washed with 1X PBS twice for 10 min and fixed with 4% paraformaldehyde for 10 min at room temperature. After fixation cultures were washed with 1X PBS three times for 10 min and stored at 4 °C until imaging was performed.

Using the Plan-Apochromat 20×/0.8 objective on Zeiss LSM 780 inverted confocal microscope and the Tile Scan function in ZEN, the entire retinal slice was imaged at high resolution. Using an original MATLAB code, the number of Zombie Red+ pixels was quantified for each image (Additional file 3). Next, using the Overlay function in the Zeiss LSM Image Browser software, the area of each retinal slice was measured. The ratio of ZR+ pixels to retinal area was determined for each projection and the Students’s t-test was performed to determine whether there was a statistically significant difference between average values at 0 h and 16 h or between laser exposed and unexposed tissue.

For in vivo labelling of RPCs in S-phase, EdU was injected intraperitoneally into P0 and P1 mice. After 6 h, the mice were sacrificed, and their eyes were harvested for cryosections. Using sharp #5 forceps, a hole was poked in the corneas and the eyes fixed with 4% paraformaldehyde for 1 h at 4 °C. After fixation, eyes were washed with 1X PBS 3 times for 10 min each. Next, eyes were incubated in 15% sucrose overnight at 4 °C and transferred to 30% sucrose for an additional night at 4 °C. Finally, the tissue was submerged in OCT compound, flash frozen, and stored at − 80 °C until sectioning. 20 μm tissue sections were obtained with a cryostat and EdU-labeled cells were detected with the Click-iT Plus EdU Alexa Fluor 555 Imaging Kit (ThermoFisher). To label RPCs in S-phase within P0 time lapse slice cultures, EdU (5 mg/mL) was added to the culture media after 6 h and this was followed by an additional 6 h in culture. Next, a small block of agarose containing the retinal slice was excised from the glass bottom dish and immediately placed in 4% paraformaldehyde to fix. The tissue was then processed, cryosectioned, and stained for EdU as described above.

For each biological replicate (*n* = 3 per group), we acquired six Z-stacks (9 μm thick) from 3 non-adjacent retinal sections. Images for quantification were obtained using the Plan-Apochromat 20X/0.8 objective and the Zeiss LSM 780 with consistent laser settings. We only imaged tissue near the optic nerve head to ensure that statistical comparisons were performed on tissue from the central retina. Finally, we generated maximum intensity projections and calculated the ratio of EdU+ pixels to DAPI+ pixels using MATLAB (Additional file 3). The average ratios for each experimental group were calculated and values were compared using a single factor ANOVA (*p* = 0.64).

### Time-lapse image acquisition and analysis

Live imaging of retinal slices was performed using the Zeiss LSM 780 inverted confocal microscope equipped with a 34-channel spectral array and 405, 458, 488, 514, 561, 594, and 633 nm laser lines or the Zeiss LSM 510 META inverted microscope equipped with a 32-channel spectral array and 405, 458, 488, 514, 543, and 633 nm laser lines. All static and live images were acquired using either a C-Apochromat 40X/1.20 W Korr. (LSM 780) or a C-Apochromat 40×/1.2 W UV-VIS-NIR objective (LSM 510).

The Zeiss LSM 780 and 510 were outfitted with an environmental chamber maintained at 37 °C and 5% CO_2_. Prior to time-lapse imaging, the retinal slice cultures were assessed by confocal microscopy and the culture with the best cross-sectional orientation was chosen for overnight imaging. Once chosen, the slice culture was allowed to equilibrate within the microscope environmental chamber for at least 30 min before initiation of image acquisition.

#### Imaging and analysis of Fucci+ RPCs

To capture cell cycle kinetics of RPCs, we cultured retinae from P0 mice expressing one or both of the Fucci reporters [41] which were excited by the 488 nm (AzG) and 561 nm (KO2) laser lines. Z-stacks with a thickness of 11.043 μm made up of 11 images were collected every 10 min. Signal intensity varied among replicates, and laser power was adjusted accordingly ranging between 2 and 4%. The Z-stack images were subsequently processed with Imaris (Bitplane) using adaptive thresholding and a Gaussian filter.

Using the spot tracking function in Imaris, we tracked the migration patterns of individual nuclei, and those data were smoothed by averaging groups of three points. The nuclear position along the apical-basal axis relative to its starting point was plotted over time for each nucleus. We tracked 56 AzG+ nuclei from 3 wildtype retinae, 77 KO2+ nuclei from 2 wildtype retinae, and 54 AzG+ nuclei from 3 *Cyclin D1*^*−/−*^ retinae.

To determine whether nuclear migration was directed or random, we calculated mean square displacement (MSD) as a function of elapsed time of individual AzG+ or KO2+ nuclear migration events [22]. These data were collected from five independent movies. MSD calculations were performed using an original MATLAB code (Additional file 4). For the AzG+ cells, the MSD was calculated from the point when an AzG+ cell first initiated apical migration (the green portion of the lines in Fig. 5b) to when it entered mitosis, during which the AzG reporter turned off. For the KO2 cells, the MSD was calculated from time zero until the time lapse movie ended or the cell left the field of view.

To compare apical nuclear velocity during G2 phase, we determined where persistent apical migration began for each nucleus and followed subsequent apical movement over the course of the time lapse. Apical velocity (V) was calculated by dividing the change in relative nuclear position by time. Using the Kolmogorov–Smirnov (KS) test, the distribution of the average and maximum velocities was determined to be non-parametric for both genotypes (V_avgWT_: *p* = 3.27 × 10^− 16^, V_avgKO_: *p* = 6.68 × 10^− 15^, V_maxWT_: *p* = 1.27 × 10^− 21^, V_maxKO_: *p* = 5.29 × 10^− 18^). A statistical difference was confirmed using a Ranksum test (V_avg_: *p* = 4.85 × 10^− 5^, V_max_: *p* = 4.71 × 10^− 8^).

#### Imaging and analysis of horizontal cells

Retinal slices from P2 *Cx57-iCre; Rosa26R-mTmG*^*+/tg*^ mice were cultured to visualize the dynamic properties of HCs during development. In all, we imaged 3 retinae for 8 to 21 h. Z-stacks with a thickness that ranged from approximately 20–27 μm made up of 19–25 images were collected every 10 min by exciting the *Rosa26R-mTmG* reporter with 488 nm (eGFP) and 561 nm (tdTomato) lasers at 4% and 2% power, respectively. For presentation purposes, movies were processed with a Gaussian filter (Imaris) or Median filter (ZEN).

Using the Filament Tracer function in Imaris, we traced the neurites of 2 adjacent HCs. For ease of viewing, we pseudo-colored (Adobe Photoshop) tracings of individual transient, vertically-oriented neurites that were previously described as being the source of repulsive homotypic interactions that establish the HC mosaic [18]. To confirm that these vertical neurites fail to interact with the neurites of neighboring HCs, we tracked them for a minimum of 360 min or until they completely retracted.

To quantify the interactions between adjacent HC neurites, neurite territory overlap was measured as previously described [30]. The HC territory was determined by assigning 10 μm diameter circular regions centered at the HC positive pixels followed by a morphological closing (dilation-erosion) process with a 10 μm diameter disk. Specifically, maximum intensity projection images were first generated from the Z-stacks at each time point. Then the HCs within each image were manually selected and separated using ImageJ. With an original MATLAB code (Additional file 5), the separated HC images underwent background removal, median filtering, and binarization. Subsequently, the HC territories were built, and we measured the area of each territory and the overlap areas between adjacent cells. Using these measurements, we calculated the overlap percentage. The delineated HC territories over time were shown with a 50% transparency overlaid on the original fluorescence images. The distance between the center of mass from the adjacent territories was also measured.

## Results and discussion

### Retinal slice culture setup

**Fig. 1:**
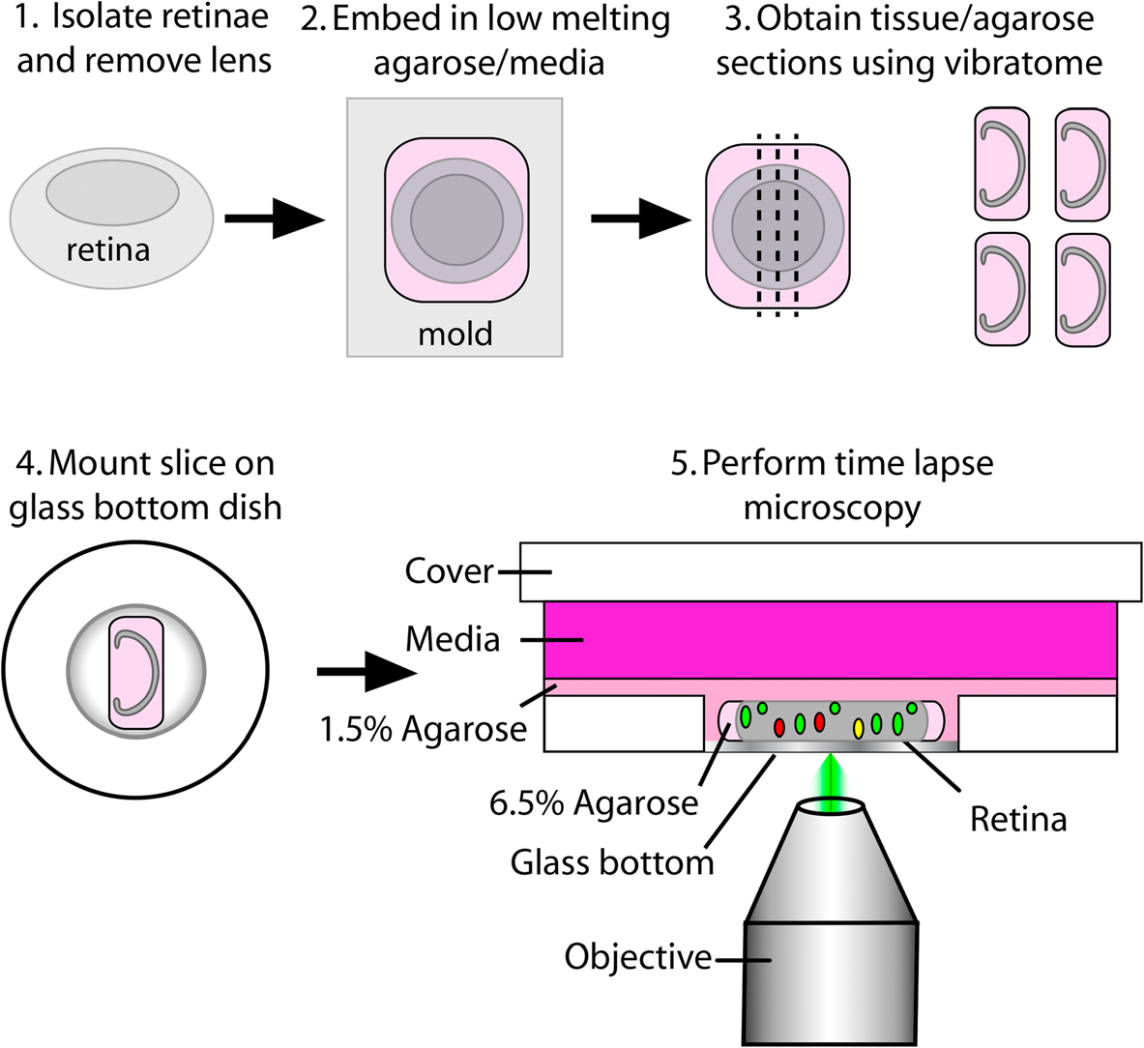
Schematic of retinal slice culture protocol

As specifically detailed in the methods section, early postnatal mouse eyes were enucleated, placed in warm culture media (DMEM/F12), and the retina was dissected away from the other ocular tissues. While this process was repeated for additional eyes, the previously isolated retinae were maintained as retinal cups submerged in DMEM/F12 and incubated at 37 °C. Once all retinae were isolated, they were then embedded in 6.5% agarose made with DMEM/F12 and 200 μm retinal cross-sections were sliced on a vibratome. Using a paintbrush, the slices were then mounted directly against the glass of a glass bottom dish and a thin layer of 1.5% agarose/media was poured over the retinal slice to maintain it against the glass. Once the agarose solidified, liquid culture media was added to the dish and the retina was ready to be imaged on an inverted microscope (summarized in Fig. 1).

### Analysis of cell death and proliferation

**Fig. 2:**
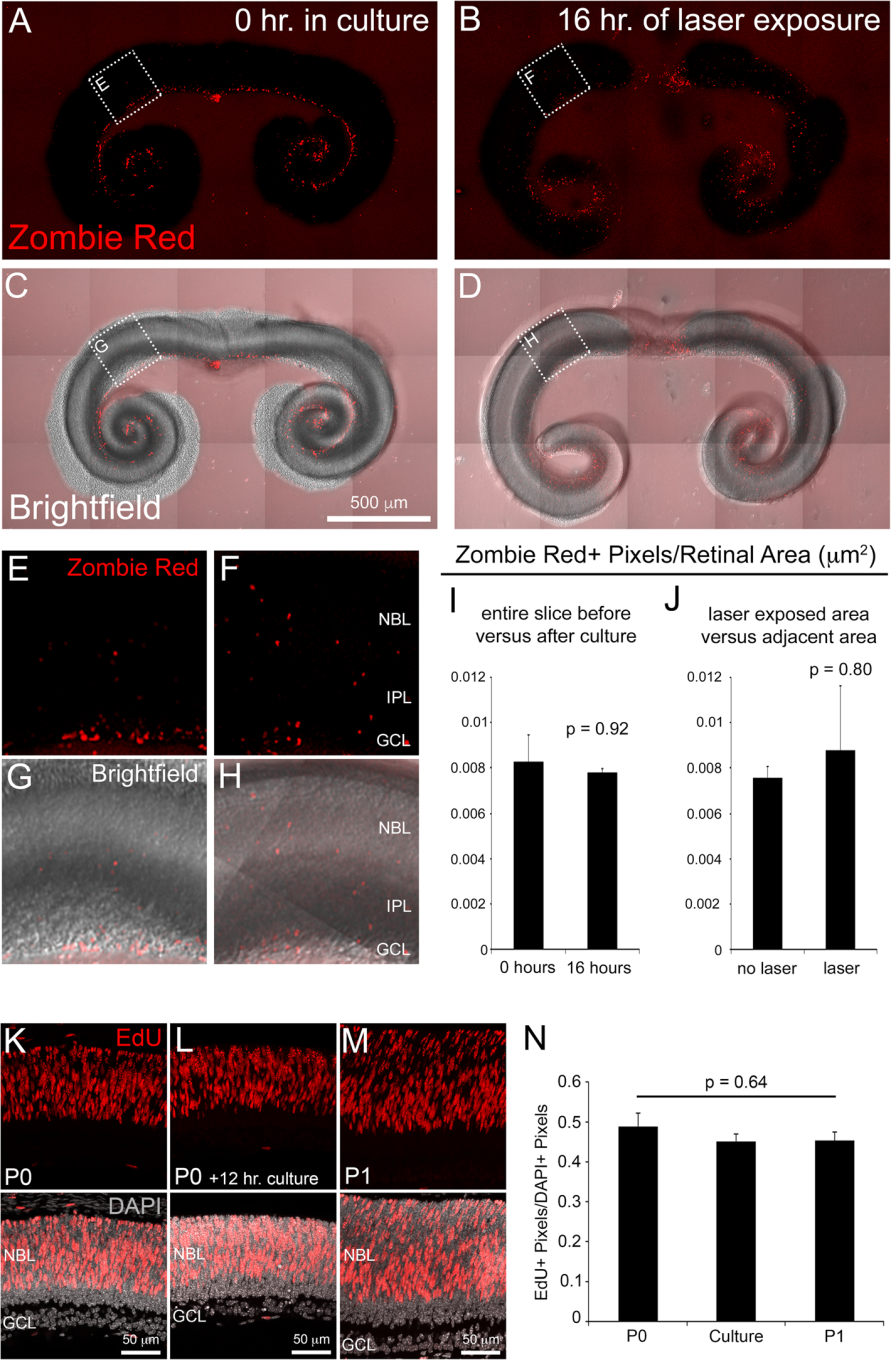
Analysis of retinal slice survival and proliferation. Retinal slice cultures stained with Zombie Red™ dye after 0 h (**a** and **c**) and 16 h (**b** and **d**) in culture. Higher magnifications of boxed region in A-D (E-H). The quantification of Zombie Red+ cells (ZR+ pixels/retinal area) showed no significant differences between 0 h and 16 h in culture (**i**) or between tissue exposed to laser versus unexposed (**j**). *n* = 9 per group. EdU labeling (6-h pulse) and quantification of RPCs in P0 and P1 retinae compared to P0 retinal slices time lapse cultures (**k**-**n**). *n* = 3 per group. Error bars represent SE. Abbreviations: NBL (neuroblastic layer), IPL (inner plexiform layer), GCL (ganglion cell layer)

To determine whether our slice culture protocol results in significant retinal cell death that would preclude meaningful analysis and interpretation, we performed Zombie Red™ staining followed by confocal imaging of fluorescence (Fig. 2a-h). Cultures of P0 retinae were stained at two time points: immediately after cultures were prepared (0 h in culture) and after 16 h of live confocal imaging (16 h of laser exposure).

Zombie Red™ (ZR) is an amine reactive fluorescent dye that is non-permeant to live cells, but permeant to cells with compromised membranes (usually dead or dying cells). The dye is optimally excited by 561 nm laser light and has a fluorescence emission peak at 642 nm (Biolegend Ca# 423109). At 0 h in culture, clear ZR+ pixels were observed throughout the ganglion cell layer (GCL) (Fig. 2a, c, e, g). ZR labeling in the GCL was entirely expected because, once the optic nerve is severed during retinal dissection, ganglion cell survival is compromised [25, 38]. Further, these data serve as a positive control for the specificity of ZR labeling of dead/dying cells.

To determine whether slice culture conditions impact RPC survival, ZR+ pixels were counted throughout the entire the neuroblastic layer (NBL) of retinal slices at 0 h and 16 h in culture and we found no significant difference (Fig. 2i). Furthermore, when we compared cell death in laser exposed tissue to an adjacent, non-laser exposed section of the same size, we observed no significant difference (Fig. 2j). These data demonstrate that, after 16 h of live imaging, the NBL of retinal slice cultures does not suffer extensive cell death that would hinder interpretation of the INM time lapse movies. Subsequent live imaging of INM, utilizing the *Fucci* reporters (see below), confirmed this conclusion as we failed to detect significant RPC nuclear fragmentation in any of our time lapse movies (Additional files 6, 7 and 8).

We next performed an assessment of RPC proliferation in our slice cultures and compared that to RPCs in vivo. Specifically, to measure the incidence of S-phase entry, P0 and P1 pups were injected with 5-ethynyl-2′-deoxyuridine (EdU) 6 h before retinae were processed for cryosections and EdU labeling. After 6 h of imaging in culture, P0 retinal slices were incubated with EdU and this was followed by an additional 6 h in culture before EdU labeling of cryosections. As expected, EdU labeling was observed throughout the NBL of the P0 and P1 retinae and the slice cultures (Fig. 2k-m). Quantification of the #EdU+/#DAPI+ pixels revealed no statistically significant decrease in S-phase entry of the slice culture versus either the P0 or the P1 time points (Fig. 2n). These data suggest that our retinal slice imaging protocol does not significantly impact RPC proliferation.

### Analysis of interkinetic nuclear migration within retinal slice cultures

As a first test of our protocol, we performed live imaging of RPC INM. INM describes the periodic movement of the nucleus within a cell that occurs in phase with cell cycle progression and is a common feature of pseudostratified epithelia such as the developing retinal neuroepithelium [5, 11, 35]. INM was first described in the neural tube of chicks and pigs [43] and has since been shown to occur in a variety of tissues in species ranging from mammals to sea anemones [15, 29, 34, 40, 46]. While this evolutionary conservation indicates importance for INM in development, its precise requirement during retinal development remains unclear. However, recent studies of the zebrafish retinae have begun to elucidate the cellular and molecular mechanisms driving INM.

**Fig. 3:**
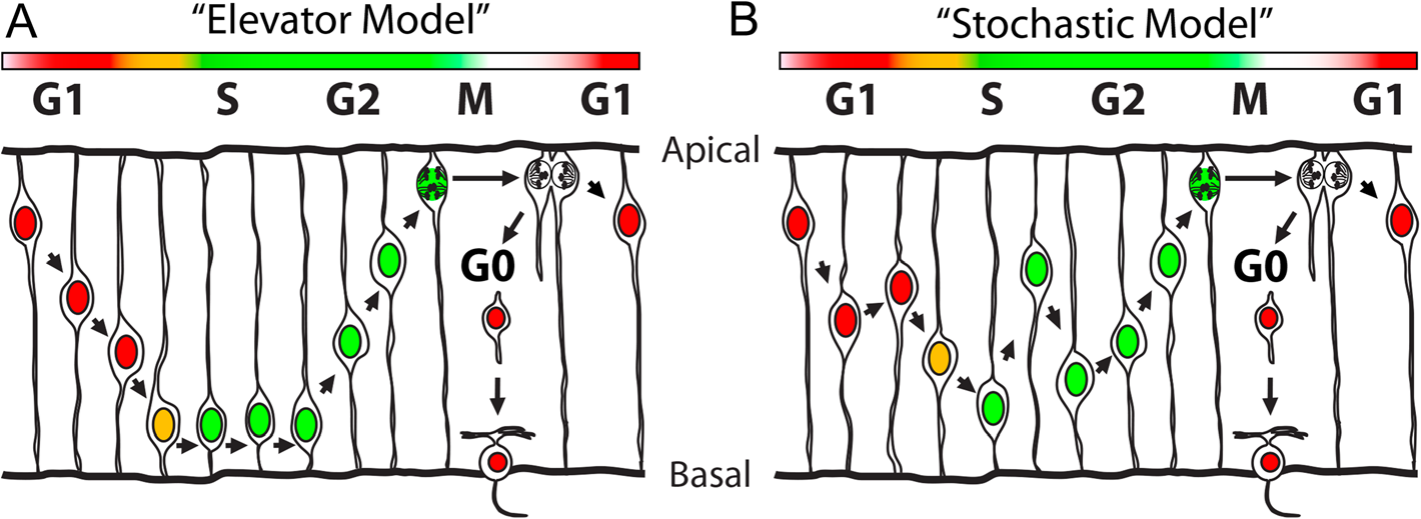
Schematic of two models of INM. The “elevator” model (**a**) and the “stochastic” model (**b**). See text for details

Initially, INM was described by an “elevator model” whereby the nuclei of cells during G1 phase undergo smooth basal migration and, upon G2 entry, nuclei migrate apically until division occurs at the apical end of the cell [43, 46] (Fig. 3a). However, previous live imaging studies performed on zebrafish retinae suggest that nuclei of cells during G1 phase migrate stochastically with a slight basal drift and S phase migration is entirely stochastic (Fig. 3b). G2 INM is indeed directed apically in the zebrafish retina prior to mitosis in the ventricular zone [22, 34]. Based on these findings, we sought to determine whether the same pattern of RPC INM occurs in the mouse.

**Fig. 4:**
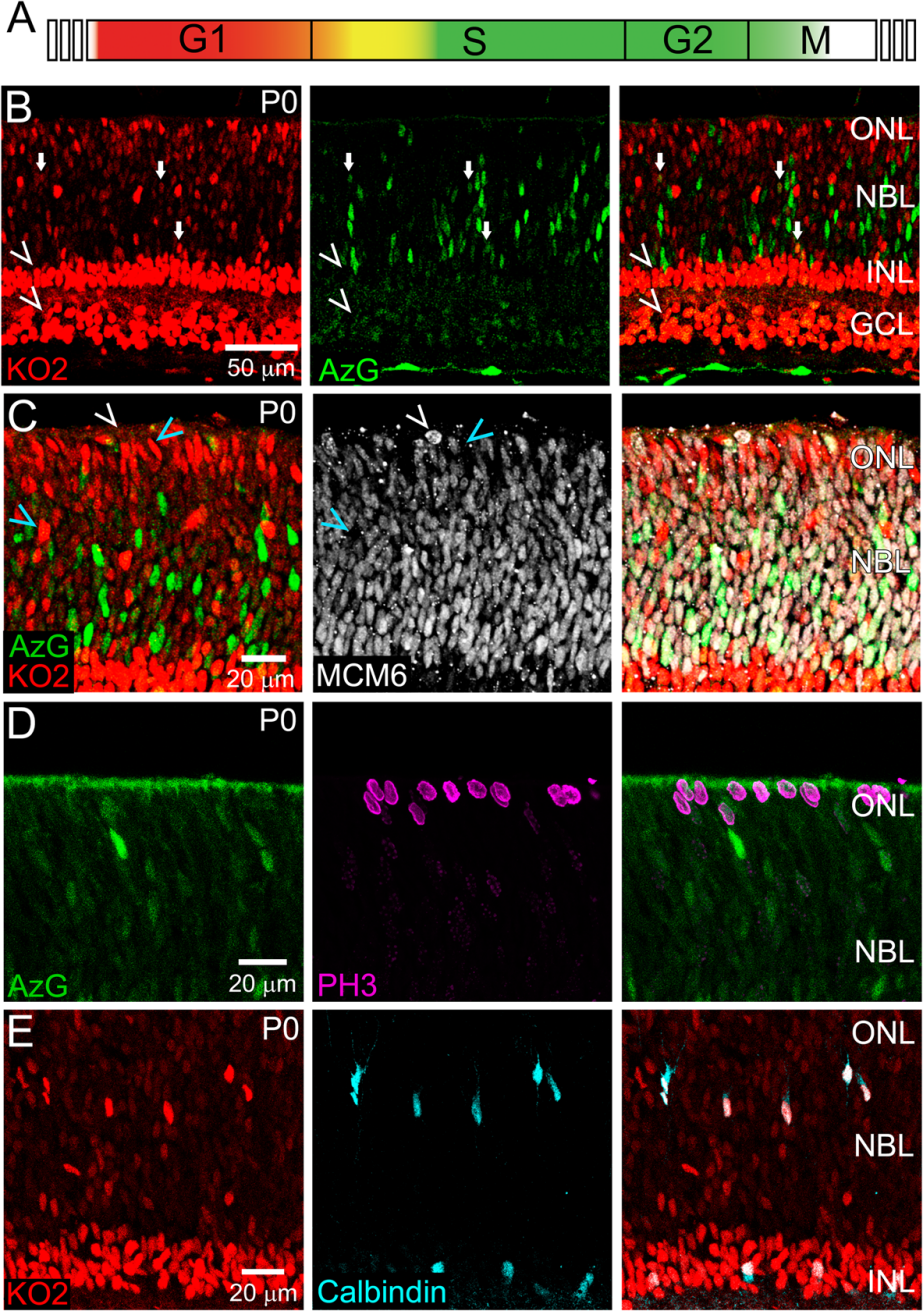
Characterization of Fucci+ cells in the P0 retina. Fucci expression throughout the cell cycle (**a**). P0 *Fucci* retinae labelled with anti-AzG (**b**), MCM6 (**c**), PH3 (**d**), and Calbindin (**e**). *n* > 3

To track INM, we utilized the *Fucci* cell cycle reporter mouse, which fluorescently labels nuclei based on cell cycle stage [41]. The *Fucci* mouse contains two transgenes each expressing a fluorescent protein. One is a variant of monomeric Kusabira Orange (KO2) and labels nuclei of cells in G1 phase. However, it has also been reported that KO2 is expressed in post-mitotic cells [41]. The other fluorescent protein is monomeric Azami Green (AzG), which labels nuclei of cells in late S and G2 phase. Cells in early S phase express both proteins (Fig. 4a). To determine precisely where the transgenes are expressed in the developing retina, we imaged fixed retinal tissue sections from *Fucci* mice. Since the AzG signal is weak after fixation, we performed immunohistochemistry using an antibody against AzG. The KO2 reporter did not require immunofluorescence for histological detection. Sections of P0 retinae revealed that KO2 is indeed expressed in the developing inner nuclear layer (INL) and GCL, which is comprised of postmitotic retinal neurons (Fig. 4b, arrowheads). As expected, the NBL is densely populated with RPCs in different stages of the cell cycle. We observed KO2+ G1 phase RPCs, AzG+ late S/G2 phase RPCs, and a small number of KO2+/AzG+ early S phase RPCs (Fig. 4b, arrows indicate KO2+/AzG+ cells).

We next performed immunofluorescence for MCM6, which labels RPCs in all stages of the cell cycle [2]. Most MCM6+ cells are co-labeled with one or both Fucci markers, with the exception of apically localized rounded nuclei preparing for division (Fig. 4c, white arrowhead). Cell cycle staging was confirmed by phospho-Histone H3 (PH3) labeling of RPC mitosis, which showed a lack of co-localization with AzG (Fig. 4d). We also noticed KO2+ cells within the presumptive HC layer and ONL that were not MCM6+ (Fig. 4c, blue arrowheads) consistent with the perdurance of KO2 in post-mitotic neurons. HC expression of KO2 was confirmed by Calbindin overlap (Fig. 4e). These cells, indicated by their KO2 brightness relative to the RPCs, were excluded from subsequent experiments that aimed to track INM within the NBL.

**Fig. 5:**
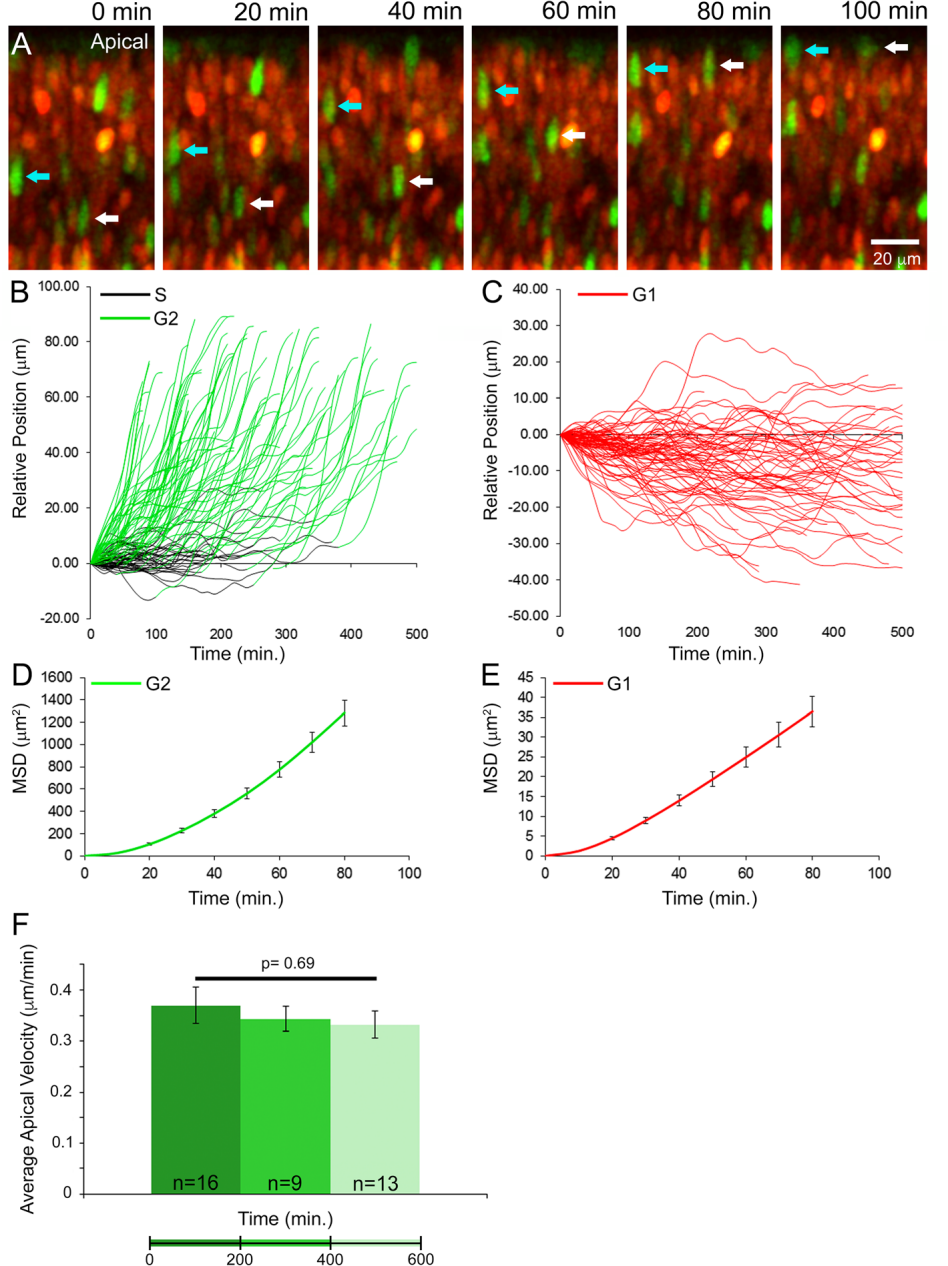
Tracking INM in the P0 retinae. Stills from time lapse movie of migrating RPC nuclei. Arrows indicate apically migrating AzG+ (G2-phase) nuclei (**a**). Relative position of nuclei over time (**b-c**). MSD of nuclei plotted over time (**d-e**). Comparison of the average apical velocity of AzG+ cells over the course of the time lapse experiments (**f**)

For culture and live confocal imaging of P0 *Fucci* retinal slices undergoing INM, Z-stacks were acquired every 10 min (Additional file 6). As shown in individual images taken from the time lapse, AzG+ cells were clearly observed migrating apically within the NBL (Fig. 5a, cyan and white arrows). With the spot tracking function in Imaris software, we tracked the migration of both AzG+ and KO2+ nuclei. All AzG+ nuclei migrated apically (Fig. 5b), but some apical migration was preceded by a period of stochastic migration, which was likely during late S phase (color-coded black in Fig. 5b). The KO2+ nuclei moved erratically, but most had an overall basal displacement (Fig. 5c). To further describe the INM patterns as random or directional, we next calculated the mean square displacement (MSD) of nuclear trajectories as a function of elapsed imaging time. For particles subject to random diffusion, the MSD is a linear function of elapsed time. When movement is directional, the MSD forms a curved line over time. When we sampled the first 80 min of each trajectory, the MSD of KO2+ and AzG+ nuclei were both nonlinear, which suggests these nuclei migrate in a directional manner (Fig. 5d and e). These findings suggest that the pattern of mouse RPC INM is similar to INM of the zebrafish retina [22, 34]. It also validates our slice culture method as being capable of capturing an essential event during mouse retinogenesis.

As an additional assessment of overall tissue health and tolerance of the live imaging protocol, we quantified the rate of G2 apical INM at different periods throughout the time lapse movies. Apical tracks of G2 nuclei were clustered into three groups (0–200 min, 200–400 min, and 400–600 min in culture) and the average apical velocity (μm/min) of nuclei was quantified for each period. We compared the average velocities among each group using a single factor ANOVA and found no statistically significant difference (Fig. 5f). Therefore, the live imaging protocol does not significantly impact RPC INM in G2 suggesting that cell cycle kinetics are not overtly affected by culture conditions or laser exposure.

### Analysis of *Cyclin D1*^*−/−*^ RPC interkinetic nuclear migration

Despite being tightly coupled to the cell cycle, chemical inhibition of INM does not cause cell cycle arrest. Rather, cells continue to proliferate, but mitosis is no longer restricted to the apical end of the ventricular zone [28, 31, 49]. In contrast, chemically blocking cell cycle progression halts INM in the rodent brain and zebrafish neuroepithelia [1, 22, 45, 47]. Additionally, studies of zebrafish mutants with an RPC cell cycle period twice as long as wild type exhibit an INM rate that is also proportionally slowed [4, 50].

Information as to how the core cell cycle machinery interfaces with the cellular mechanisms driving INM is only beginning to emerge. The most established role is for Cyclin dependent kinase 1 (CDK1) which, along with CYCLIN A or CYCLIN B, is well-known to regulate the entry into G2 and progression toward M-phase [24, 37]. Pharmaceutical inhibition of cdk1 in zebrafish resulted in RPCs that stalled in G2 and failed to undergo apical INM suggesting that cdk1 is necessary for INM [45]. Remarkably, inhibition of the cdk1 inhibitor wee1 resulted in precocious cdk1 activity and apical migration of RPCs that are presumably in S-phase. Thus, cdk1 activity is both necessary and sufficient for apical INM [45]. It is not yet clear whether a similar role for CDK1 exists during mouse INM or whether other G2-specific genes directly drive INM.

The best, in vivo-characterized mouse RPC cell cycle regulator is CYCLIN D1 [9, 10, 13, 44]. Although its canonical role is to promote S phase entry, CYCLIN D1 is expressed throughout the cell cycle in RPCs, and its loss extends retinal cell cycle length [2, 9, 10]. Specifically, these studies used thymidine analog incorporation and antibody staining in fixed mouse retinal tissue to demonstrate that the length of G1, G2, and M phases combined was extended in the absence of *Cyclin D1*. Given the tight coupling of cell cycle timing and INM rate, we hypothesized that apical migration during G2 phase in *Cyclin D1*^*−/−*^ (KO) retinae would be slower than in wildtype (WT) retinae.

**Fig. 6:**
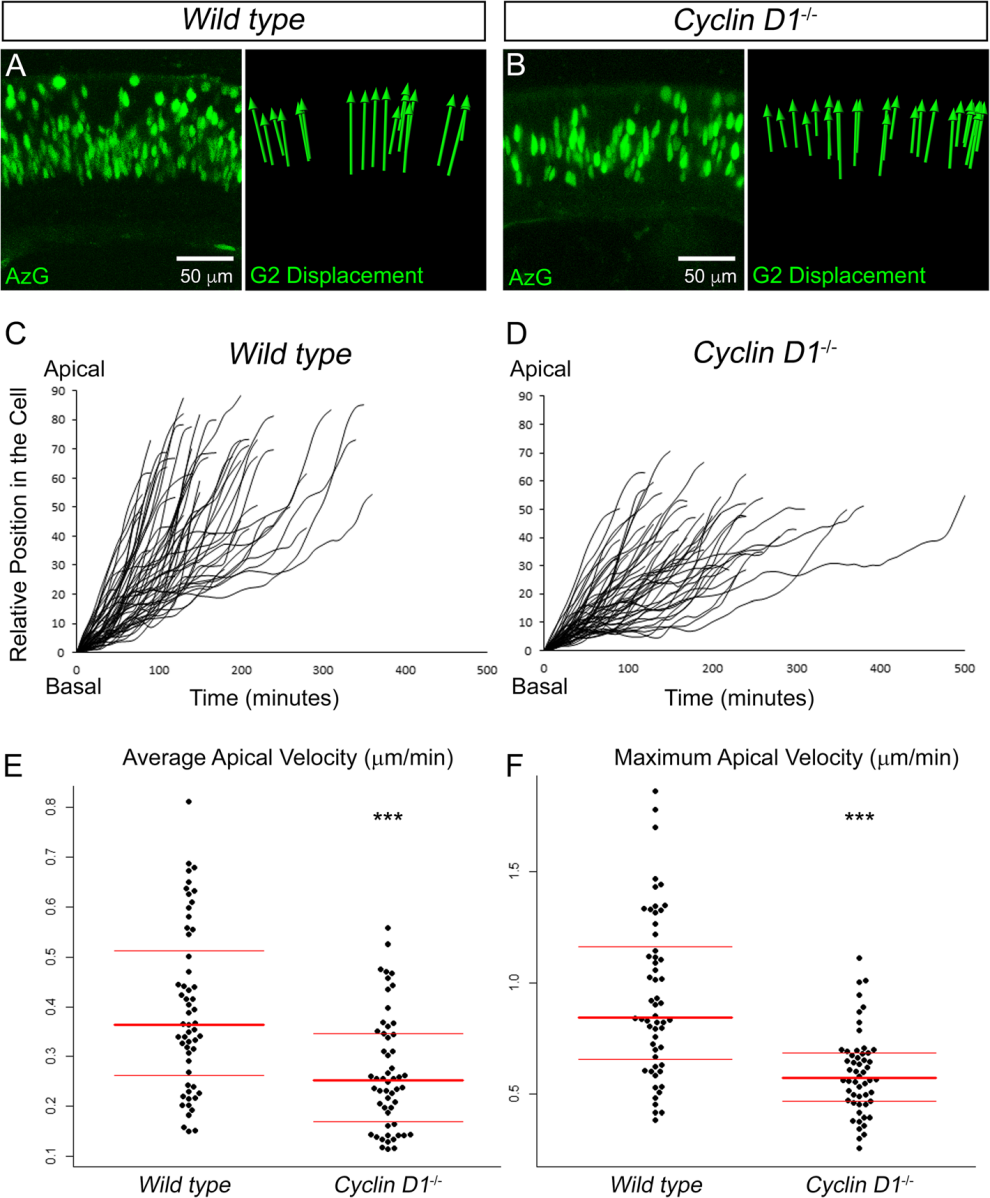
Apical migration in the *Cyclin D1*^*−/−*^ retina. Still images from time lapse movies of migrating wild type and mutant AzG+ RPCs and the overall displacement of each nucleus (**a-b**). Relative position of nuclei over time (**c-d**). The distributions of average and maximum apical velocities of tracked nuclei (**e-f**). Data were collected from 56 nuclei in 3 WT retinae and 54 nuclei in 3 KO retinae. ***p < 0.001

To test this, we imaged (at least 12 h per retina) WT (*n* = 3) and KO (*n* = 3) retinae expressing AzG (Additional files 7 and 8). Both WT and KO AzG+ RPCs exhibited the expected apical displacement characteristic of G2 phase (Fig. 6a-b). Using the spot tracking function in Imaris, we tracked the position of 56 AzG+ nuclei from WT RPCs and 54 nuclei from KO RPCs over time. As mentioned previously, AzG labels nuclei of cells during late S and G2 phase. Thus, to analyze G2 apical migration more specifically, we omitted data points prior to the first indication of directed apical migration and plotted the relative nuclear position over time (Fig. 6c-d). We found that AzG+ nuclei in the KO retinae had slower average and slower maximum apical velocities (Fig. 6e-f). Taken together with evidence from previous studies [9, 10], these data suggest that CYCLIN D1 may indirectly regulate INM rate by maintaining cell cycle timing during G2 phase and support conclusions from zebrafish studies that suggest INM rate and cell cycle length are coupled [4, 50].

### Analysis of horizontal cell dynamics

**Fig. 7:**
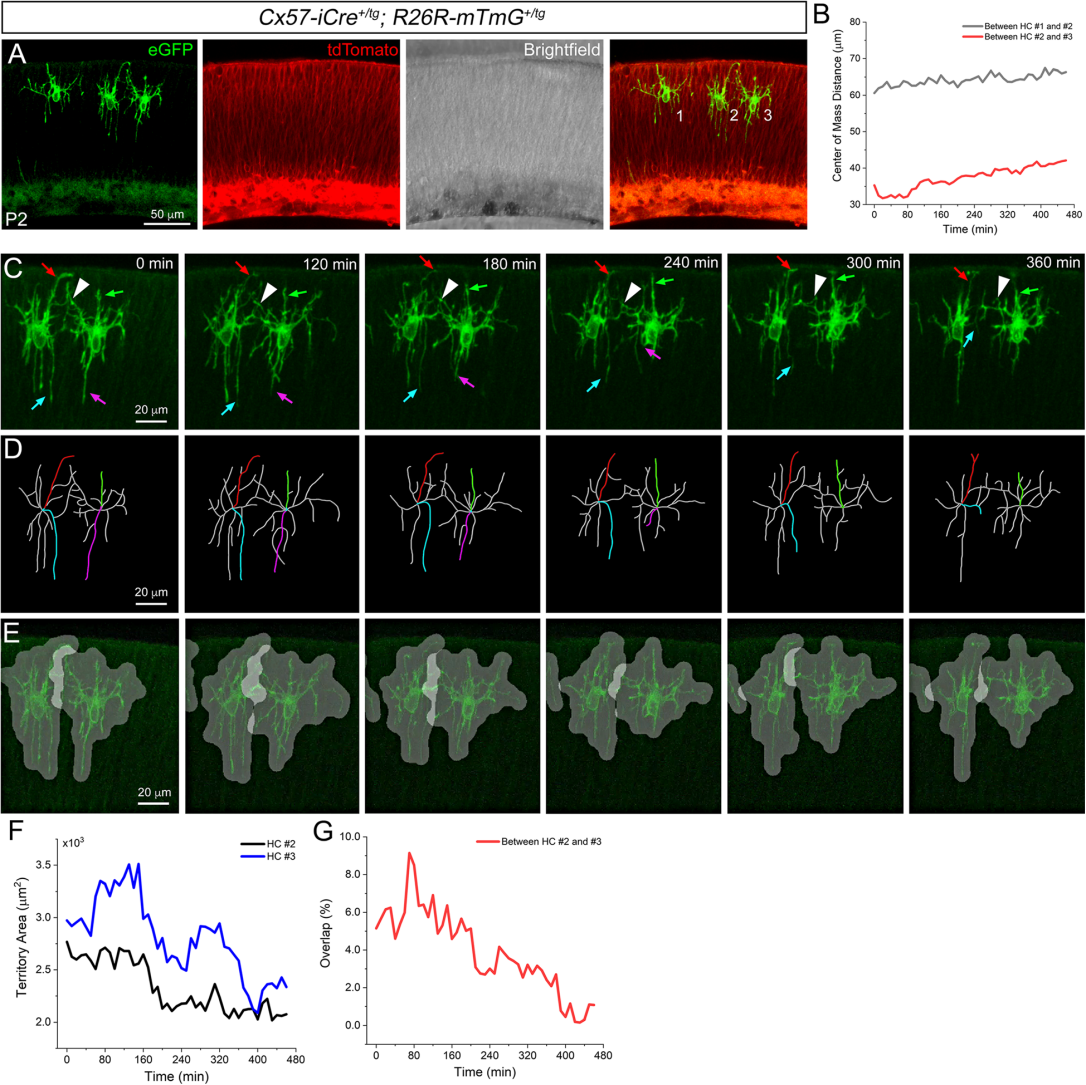
Live imaging of horizontal cells. Still image from a time lapse movie of a P2 *Cx57-iCre*^*+/tg*^*; Rosa26R-mTmG*^*+/tg*^ retina (**a**). Center of mass distance measurement indicating little HC somal translocation (**b**). Selected images series from a time lapse movie of two horizontal cells (**c**). The colored arrows indicate dynamic, vertically-oriented neurites that do not overlap with neurites of the adjacent cell. The white arrowheads indicate more laterally-oriented neurites that exhibit overlap with the adjacent cell. Tracings of HC neurites with selected vertical neurite traces colored (**d**). Highlighted territories of overlap between adjacent HCs (**e**). Measurements of total HC area (**f**) and neurite overlap over time (**g**). n > 3 movies

Finally, we sought to determine whether our live imaging protocol is suitable for capturing discrete developmental events among retinal neurons. As a proof-of-concept, we imaged early postnatal horizontal cell (HC) dynamics. We generated *Cx57-iCre*^*+/tg*^*; Rosa26R-mTmG*^*+/tg*^ mice [16, 32] in which early postnatal HCs express a membrane-bound green fluorescent protein (eGFP) that clearly highlights HC neurites. The remainder of the retina, without Cre-mediated recombination, is labeled with a membrane-bound tdTomato fluorescent protein (Fig. 7a).

Live imaging of HCs at P2 revealed soma within the presumptive HC layer that showed little movement whereas the neurites were extremely dynamic (Fig. 7a-b and Additional files 9, 10 and 11). Specifically, the more laterally-oriented neurites of adjacent HCs exhibited extension and retraction toward one another and generally maintained significant overlap (Fig. 7c, arrowheads). In contrast, vertically-oriented neurites of the same HCs underwent continuous extension and retraction but appeared to exhibit little or no overlap with the neighboring cell (Fig. 7c, colored arrows and Fig. 7d, colored filaments). To further analyze this cellular behavior, we next measured the territorial overlap between neurites of adjacent HCs (Fig. 7e) [30]. The HC territory over time was determined followed by the overlap of those areas between adjacent cells (Fig. 7f-g). In order to visually highlight territories of overlap, the delineated HC territories over time were shown with a 50% transparency overlaid on the original fluorescence images. Thus, the brighter white regions, covering more horizontally-oriented neurites, indicate overlap whereas we did not detect overlap between vertical neurites (Fig. 7e and Additional file 12). Previous live, multiphoton microscopy of mouse HCs in explant cultures identified these vertical neurites as transient processes that mediate homotypic repulsive interactions driving HC mosaic formation [18].

## Conclusions

Live imaging of developing retinal slices is capable of directly monitoring and quantifying important events during mouse retinogenesis. This method is compatible with a variety of imaging systems and fluorescent reporters thereby making live retinal imaging a more practical option for researchers without access to 2-photon microscopy. The main limitation of this approach is the length of time one can culture and image a retinal slice. In our hands, by 48 h, tissue artifacts were present in the retina and obvious cell blebbing occurred. In the future, by employing conditioned media and other growth factors, we may be able to extend the life of the slice culture. However, several dynamic developmental processes, as demonstrated in this study, can be readily imaged for shorter periods (up to at least 16 h) without obvious signs of significant cell death. Furthermore, the cross-sectional view provided by the retinal slice provides excellent resolution of the entire retinal thickness. As an example, using the *Mito-Dendra*^*+/tg*^ mouse, we have recently generated movies of sufficient resolution to capture mitochondrial dynamics within the developing retina [36] (Additional files 13 and 14).

While not explicitly demonstrated in this study, for certain cell types and developmental processes, retinal slice cultures may also circumvent the need for confocal microscopy and commercial environmental chambers. Previously, mouse retinal whole mount explants were shown to develop under CO_2_-independent culture conditions [33]. Therefore, one may be able to simply construct a homemade heater box [19] surrounding the stage of an epifluorescent microscope.

## Additional files

Additional file 1: List of materials used for retinal slice culture preparation and imaging. (DOCX 23 kb)

Additional file 2: List of antibodies used for immunofluorescence. (DOCX 17 kb)

Additional file 3: Code written for MATLAB to quantify Zombie Red+ and EdU+ pixels (TXT 1 kb)

Additional file 4: Code written for MATLAB to calculate mean square displacement (MSD). (TXT 849 bytes)

Additional file 5: Code written for MATLAB to calculate HC neurite territory overlap. (TXT 6 kb)

Additional file 6: Time lapse movie of P0 Fucci+ RPCs undergoing INM. Individual nuclei were tracked using Imaris software. Z-stacks with a thickness of 14.4 μm made up of 6 images were collected every 10 min for 650 min. Playback: 10 frames per second. (MOV 4776 kb)

Additional file 7: Time lapse movie of wild type P0 RPCs in undergoing apical migration during G2-phase (AzG+) of the cell cycle. Z-stacks with a thickness of 14.4 μm made up of 6 images were collected every 10 min for 750 min. Playback: 10 frames per second. (MOV 15874 kb)

Additional file 8: Time lapse movie of *Cyclin D1*^*−/−*^ P0 RPCs in undergoing apical migration during G2-phase (AzG+) of the cell cycle. Z-stacks with a thickness of 14.4 μm made up of 6 images were collected every 10 min for 700 min. Playback: 10 frames per second. (MOV 11565 kb)

Additional file 9: Time lapse movie of P2 horizontal cell dynamics. Z-stacks with a thickness of 19.90 μm made up of 19 images were collected every 10 min for 460 min. Playback: 10 frames per second. (MOV 14592 kb)

Additional file 10: Time lapse movie of P2 horizontal cell dynamics. Z-stacks with a thickness of 19.90 μm made up of 19 images were collected every 10 min for 460 min Playback: 10 frames per second. (MOV 585 kb)

Additional file 11: Time lapse movie of P2 horizontal cell dynamics. Z-stacks with a thickness of 23.2 μm made up of 22 images were collected every 10 min for 1240 min. Playback: 10 frames per second. (MOV 1788 kb)

Additional file 12: Time lapse movie of P2 horizontal cell neurite overlap. Z-stacks with a thickness of 19.90 μm made up of 19 images were collected every 10 min for 460 min. Playback: 10 frames per second. (MOV 1849 kb)

Additional file 13: Time lapse movie of mitochondrial dynamics within a P0 retina. Z-stacks with a thickness of 14.4 μm made up of 6 images were collected every 10 min for 530 min. Playback: 15 frames per second. (MOV 49960 kb)

Additional file 14: High magnification time lapse movie of the same retina in Additional file 13 imaged for an additional 390 min. Playback: 15 frames per second. (MOV 39725 kb)

## Abbreviations

AzG: Azami Green
FBS: Fetal Bovine Serum
GCL: Ganglion Cell Layer
HC: Horizontal Cell
INL: Inner Nuclear Layer
INM: Interkinetic Nuclear Migration
KO2: Kusabira Orange
MSD: Mean Square Displacement
NBL: Neuroblastic Layer
ONL: Outer Nuclear Layer
RPC: Retinal Progenitor Cell
