# Supplementary material for: Live imaging of developing mouse retinal slices
Source: Neural Dev. 2018 Sep 15;13:23. doi: 10.1186/s13064-018-0120-y (PMC6139133; doi:10.1186/s13064-018-0120-y)
Supplement: Supplementary file 1 — List of materials used for retinal slice culture preparation and imaging. (DOCX 23 kb) [file 13064_2018_120_MOESM1_ESM.docx]

| **Reagent**  **TABLE S1. List of materials used for retinal slice culture preparation and imaging.** | **Source** | **Comments** |
| --- | --- | --- |
| **Agarose Solutions** |  |  |
| 50mL Conical centrifuge tubes |  | Generic brand |
| DMEM/F12 media | Thermo Fisher Scientific  (11039021) |  |
| GeneMate low melt agarose | BioExpress  (E-3126-25) |  |
| Pyrex® 1L beaker | Corning, Inc. |  |
| Pyrex® 600mL beaker | Corning, Inc. |  |
| Isotemp™ basic stirring hotplate | Thermo Fisher Scientific |  |
| **Culture Media** |  |  |
| 50mL Conical centrifuge tubes |  | Generic brand |
| DMEM/F12 | Thermo Fisher Scientific  (11039021) |  |
| Penicillin/Streptomycin (Pen/Strep) | Gibco  (15140122) |  |
| Fetal bovine serum (FBS) | GenDEPOT, Inc.  (F0900-050) |  |
| Insulin, human recombinant, zinc solution | Thermo Fisher Scientific  (12585-014) | 4mg/ml stock solution |
| Heratherm™ microbiological incubator | Thermo Fisher Scientific  (IGS60) | Set at 37°C |
| **Dissection** |  |  |
| Small petri dish | Corning, Inc.  (351008) |  |
| Large petri dish | Corning, Inc.  (351029) |  |
| SnuggleSafe® microwaveable heatpad | Lenric C21 Ltd |  |
| Microwave | Danby Designer  (DMW077BLSD) |  |
| Zeiss Stemi 2000 microscope | Carl Zeiss, Inc. |  |
| Fine scissors | Fine Science Tools  (FST 14040-10) |  |
| Graefe forceps | Roboz Surgical Instrument Co.  (RS-5130) |  |
| Curved Graefe serrated forceps | Fine Science Tools  (FST 11052-10) |  |
| Dumont #5 fine forceps | Fine Science Tools  (FST 11254-20) |  |
| 70% EtOH | Decon Labs, Inc.  (2401) |  |
| Dissection pads | VWR  (56617-018) |  |
| 1X Phosphate Buffered Saline (PBS) | GenDEPOT  (P2100-050) | 10X Stock |
| Stereo microscope fluorescence adaptor | Nightsea | Royal Blue and Green adaptors were used |
| **Embedding** |  |  |
| Tissue-Tek® cryomold | Sakura  (4565) |  |
| Plastic transfer pipette |  | Generic brand |
| Kimwipes™ delicate task wipers | Kimberly-Clark Professional  (34155) |  |
| **Mounting, Culturing, and Sectioning** |  |  |
| Vibratome | Leica Biosystems  (VT1000S) |  |
| 1X PBS | GenDEPOT  (P2100-050) | 10X Stock |
| Double-edged razor blade | Personna American Safety Razor Company |  |
| AMF hexagon screwdriver | Matoopa  (906-3) |  |
| Value bright pocket LED flashlight | Rayovac |  |
| Paint brush | Dynasty  (2157R) | Size 3 |
| Single edge industrial razor blade | VWR  (55411-055) |  |
| Plastic transfer pipette |  | Generic brand |
| Super glue ultra-gel control^TM^ | Loctite®  (43903) |  |
| Glass bottom culture dish | MatTek Corporation  (P35G-0-10-C) | Can be reused after rinsing thoroughly with 70% EtOH |
| P1000 pipette and tips |  | Generic brand |
| P200 pipette and tips |  | Generic brand |
| Forma Series II water jacket CO_2_ incubator | Thermo Fisher Scientific  (3110) | Set at 37°C and 5% CO_2_ |
| **Imaging** |  |  |
| Zeiss LSM 780 inverted confocal microscope | Carl Zeiss, Inc. | Outfitted with incubation chamber for regulating temperature and CO_2_ |
| Zeiss LSM 510 inverted confocal microscope | Carl Zeiss, Inc. | Outfitted with incubation chamber for regulating temperature and CO_2_ |
| Immersol W 2010 | Carl Zeiss, Inc.  (444969-0000-000) |  |
