# Supplementary material for: Live imaging of developing mouse retinal slices
Source: Neural Dev. 2018 Sep 15;13:23. doi: 10.1186/s13064-018-0120-y (PMC6139133; doi:10.1186/s13064-018-0120-y)
Supplement: Supplementary file 2 — List of antibodies used for immunofluorescence. (DOCX 17 kb) [file 13064_2018_120_MOESM2_ESM.docx]

| **Primary Antibody** | **Host Species** | **Dilution** | **Source** | **Secondary Antibody** | **Source** |
| --- | --- | --- | --- | --- | --- |
| Anti-Calbindin | Rabbit | 1:1,000 | Swant (CB38) | goat-α-rabbit 647 | Thermo Fisher Scientific  (A-21245) |
| Anti-MCM6 | Goat | 1:200 | Santa Cruz (sc-9843) | donkey-α-goat 647 | Thermo Fisher Scientific  (A-21447) |
| Anti-Monomeric Azami-Green 1 | Rabbit | 1:400 | MBL (PM052M) | goat-α-rabbit 488 or  donkey-α-rabbit 488 | Thermo Fisher Scientific  (A-11034)  or  (A-21206) |
| Anti-Phospho-Histone H3 | Rabbit | 1:500 | Millipore (06-570) | goat-α-rabbit 647 | Thermo Fisher Scientific  (A-21245) |

**TABLE S2. List of antibodies used for immunofluorescence.**
